# Circadian Clock Regulation of the Cell Cycle in the Zebrafish Intestine

**DOI:** 10.1371/journal.pone.0073209

**Published:** 2013-08-27

**Authors:** Elodie Peyric, Helen A. Moore, David Whitmore

**Affiliations:** Centre for Cell and Molecular Dynamics, Department of Cell and Developmental Biology, University College London, London, United Kingdom; Morehouse School of Medicine, United States of America

## Abstract

The circadian clock controls cell proliferation in a number of healthy tissues where cell renewal and regeneration are critical for normal physiological function. The intestine is an organ that typically undergoes regular cycles of cell division, differentiation and apoptosis as part of its role in digestion and nutrient absorption. The aim of this study was to explore circadian clock regulation of cell proliferation and cell cycle gene expression in the zebrafish intestine. Here we show that the zebrafish gut contains a directly light-entrainable circadian pacemaker, which regulates the daily timing of mitosis. Furthermore, this intestinal clock controls the expression of key cell cycle regulators, such as *cdc2*, *wee1*, *p21, PCNA* and *cdk2*, but only weakly influences *cyclin B1, cyclin B2* and *cyclin E1* expression. Interestingly, food deprivation has little impact on circadian clock function in the gut, but dramatically reduces cell proliferation, as well as cell cycle gene expression in this tissue. Timed feeding under constant dark conditions is able to drive rhythmic expression not only of circadian clock genes, but also of several cell cycle genes, suggesting that food can entrain the clock, as well as the cell cycle in the intestine. Rather surprisingly, we found that timed feeding is critical for high amplitude rhythms in cell cycle gene expression, even when zebrafish are maintained on a light-dark cycle. Together these results suggest that the intestinal clock integrates multiple rhythmic cues, including light and food, to function optimally.

## Introduction

The circadian clock is a self-sustained endogenous oscillator that generates daily rhythms in behavior and physiology with a period of approximately 24 hours, even in absence of external cues [1]. Synchronizing this clock to the environmental light-dark cycle is thought to provide a survival advantage by allowing organisms to predict environmental changes and optimize the relative timing of their behavior and internal physiology [2,3]. A variety of physiological processes are regulated by the circadian clock, including the sleep-wake cycle, body temperature, feeding behavior, metabolism, cell cycle progression and gastrointestinal function. Major digestive activities display a daily rhythm, including motility, maintenance and replacement of the protective epithelial barrier, nutrient absorption and production of digestive enzymes [4,5]. Of particular importance is the fact that intestinal epithelial cells exhibit rhythmic cell division, differentiation and apoptosis [6–9].

The classical view of circadian clock organization in the majority of animal species was one of a central, master pacemaker, either in the suprachiasmatic nucleus (SCN) of mammals, or in the eyes and pineal gland of lower vertebrates. This view has changed dramatically over the years, with considerable evidence for independent circadian oscillators within numerous, if not all, peripheral tissues. In mammals, this includes the presence of peripheral clocks in digestive tissues, including pancreas, liver, stomach and intestine [9–12]. Circadian clock organization in zebrafish is even more decentralized than in mammals, as most zebrafish tissues not only possess an endogenous clock, but also are directly light responsive [13,14]. Despite this fact, the presence and function of peripheral clocks in the zebrafish gastrointestinal tract remains largely unexplored.

The renewal of cells within the intestine is a critical aspect of its physiology. In mammals, new cells are generated from a stem cell population found at the base of the intestine in crypts, before differentiating and migrating along the length of the intestinal villi [15]. The timing of this cell division is under the control of the circadian clock, and clock genes have been shown to oscillate throughout the mouse intestinal tract [12]. However, relatively little is known about how the clock regulates cell cycle timing or which specific cell cycle genes might be under direct clock control in this particular tissue. In addition, entrainment of the intestinal clock in mammals appears to be quite complicated, with systemic signals from the central clock in the SCN playing a role, in coordination with local cellular clocks, as well as entraining signals occurring directly from the ingestion of food [16]. How these signals are then integrated to control cell cycle timing and gene expression in the gut is not yet clear. Such an understanding is of clear clinical importance given the overwhelming evidence that disruption of circadian clock function can lead to an increased risk of cancer [17,18].

To explore these issues further, we have examined circadian clock function in adult zebrafish gut. We monitored the daily timing of cell division and identified several cell cycle genes that are under clock control. Although the circadian system in zebrafish is highly decentralized, the presence of a circadian clock has never been established in adult intestine, nor has the existence of clock-controlled cell cycle progression. Zebrafish, therefore, represent a novel model system with which to study this aspect of intestinal function and physiology. Examination of rhythmic cell cycle gene expression in the gut might provide clues to the mechanism by which clock-cell cycle regulation occurs. Furthermore, the direct light sensitivity of zebrafish tissues allows us to explore entrainment of the intestinal clock to light, as well as to food. The impact and integration of both of these cues on clock-cell cycle regulation will be determined. Finally, we will investigate the consequences of food deprivation on both circadian clock function, as well as cell proliferation in the intestine.

## Results

### Adult zebrafish intestine possesses a directly light-responsive, circadian pacemaker

**Figure 1:**
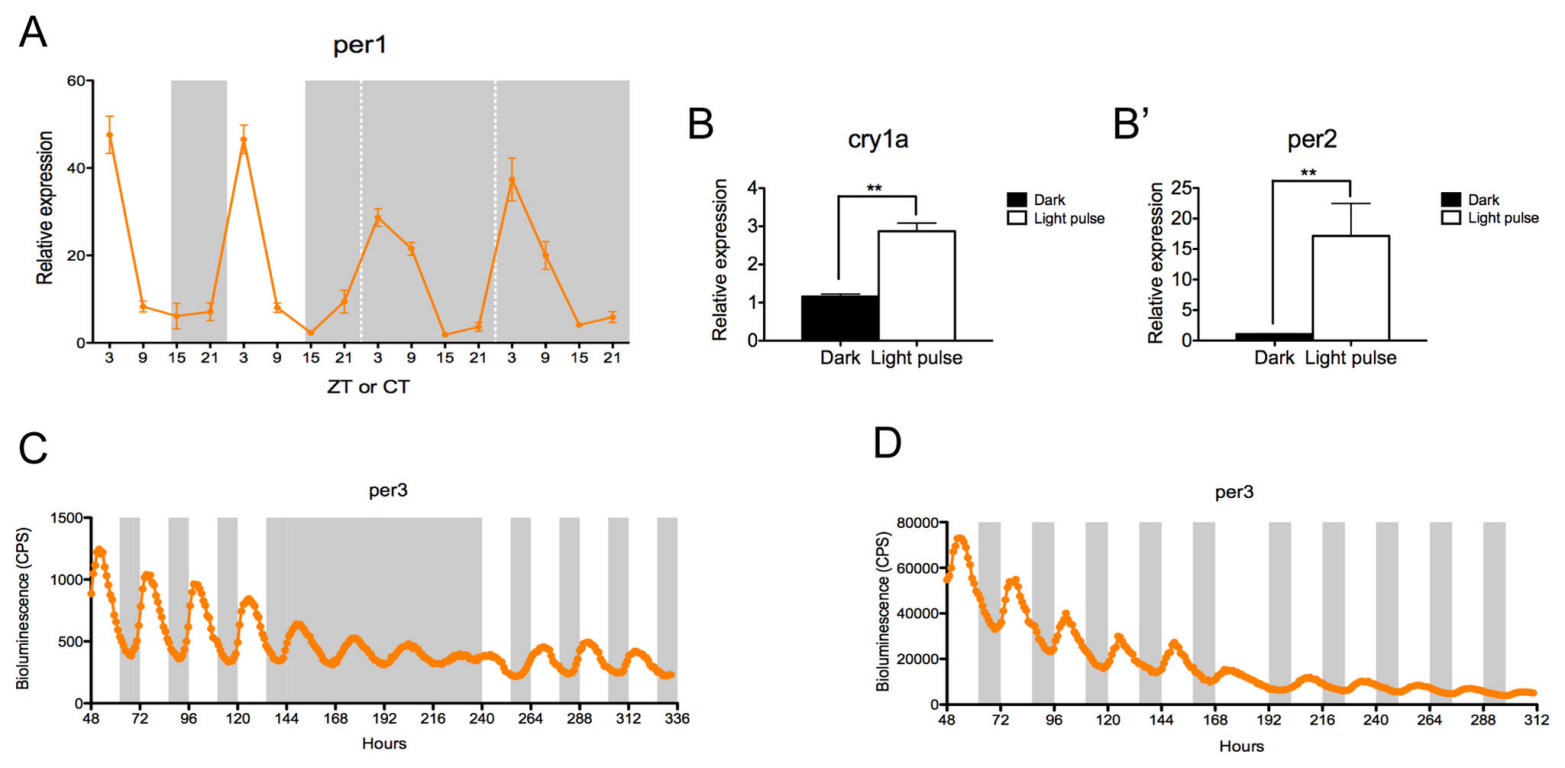
Zebrafish intestine possesses a directly light-responsive circadian pacemaker. (A) After entrainment to a LD cycle (14L:10D), the expression of the core clock component *per1* is rhythmic in the intestine with a peak at ZT3. The oscillation is maintained when animals free-run in DD. Data represents the mean ± SEM from 8 fish per indicated zeitgeber or circadian time (ZT or CT), where ZT0 is lights on. (B–B’) A three-hour light pulse induces expression of *cry1a* and *per2* compared to a dark control. Data represent the mean ± SEM from 5 fish. (C) The adult intestine of *per3-luciferase* zebrafish entrained to 4 days of LD, 4 days of DD and returned to 4 days in LD was monitored. Gut *per3* expression is rhythmic in LD with a peak at ZT5 and free-runs in DD with a damped amplitude. The mean bioluminescence in counts per seconds (CPS) is plotted (n=3-4). (D) Intestine of adult *per3-luciferase* zebrafish were entrained to 5 days of LD then transferred in DL for 6 days. Gut *per3* is able to re-entrain to a new, reversed light regime. The mean bioluminescence in CPS is plotted (n=3-4). White and grey backgrounds represent light and dark phases, respectively.

To determine if the zebrafish gut contains an endogenous circadian pacemaker, adult zebrafish were maintained on a LD cycle of 14 hours of light, 10 hours of dark (14L: 10D) and fed twice a day. Fish were sacrificed at 6-hour intervals over a period of 4 days. Gut samples were collected for two days on a light-dark (LD) cycle, and then for a further two days in constant darkness (DD), in order to show that any oscillations were due to an endogenous clock and not driven by the environmental LD cycle. Samples were analyzed by quantitative RT-PCR (qPCR) to determine the expression levels of *per1*. This gene represents a key element of the core clock mechanism and in most teleosts studied to date, shows high amplitude circadian oscillations [19]. In LD, *per1* expression in the gut displays a strong circadian rhythm with a peak at zeitgeber time (ZT) 3 (where ZT0 represents lights-on). This oscillation continues robustly as the fish free-run into DD, demonstrating the clear presence of an intestinal circadian pacemaker (Figure 1A). The intestine also appears to be light responsive, as a three-hour light pulse given at circadian time (CT) 16 to fish maintained in constant darkness induces expression of both *cry1a* and *per2*, two genes believed to be critical for light entrainment in zebrafish (Figure 1B) [20–22].

Not surprisingly, the circadian clock functions in the gut *in vivo*, but to demonstrate that this clock is endogenous, we made use of the transgenic *period3* (*per3*)*-luciferase* fish and *in vitro* tissue culture approaches [23]. Bioluminescent traces of intestinal tissue from *per3-luciferase* fish reveals high amplitude rhythms of *per3* expression on a LD cycle, with a peak at ZT5 and a period of 24.0 ± 0.4 hours (Figure 1C). In DD, *per3* expression remains rhythmic with an average period of 26.1 ±0.1 hours (mean ± SEM). When the cultures are returned to a LD regime, *per3* rhythms are re-established with a peak at ZT5 (Figure 1C). To demonstrate the direct light sensitivity of the zebrafish intestine, tissues entrained to a LD cycle were then exposed to a reversed LD cycle, 12 hours out of phase. The waveform of *per3* expression acutely alters during re-entrainment, but within one circadian cycle, the cultured intestines have now stably re-entrained to the new, reversed LD cycle (Figure 1D).

### M phase of the cell cycle is rhythmic and under circadian clock control

**Figure 2:**
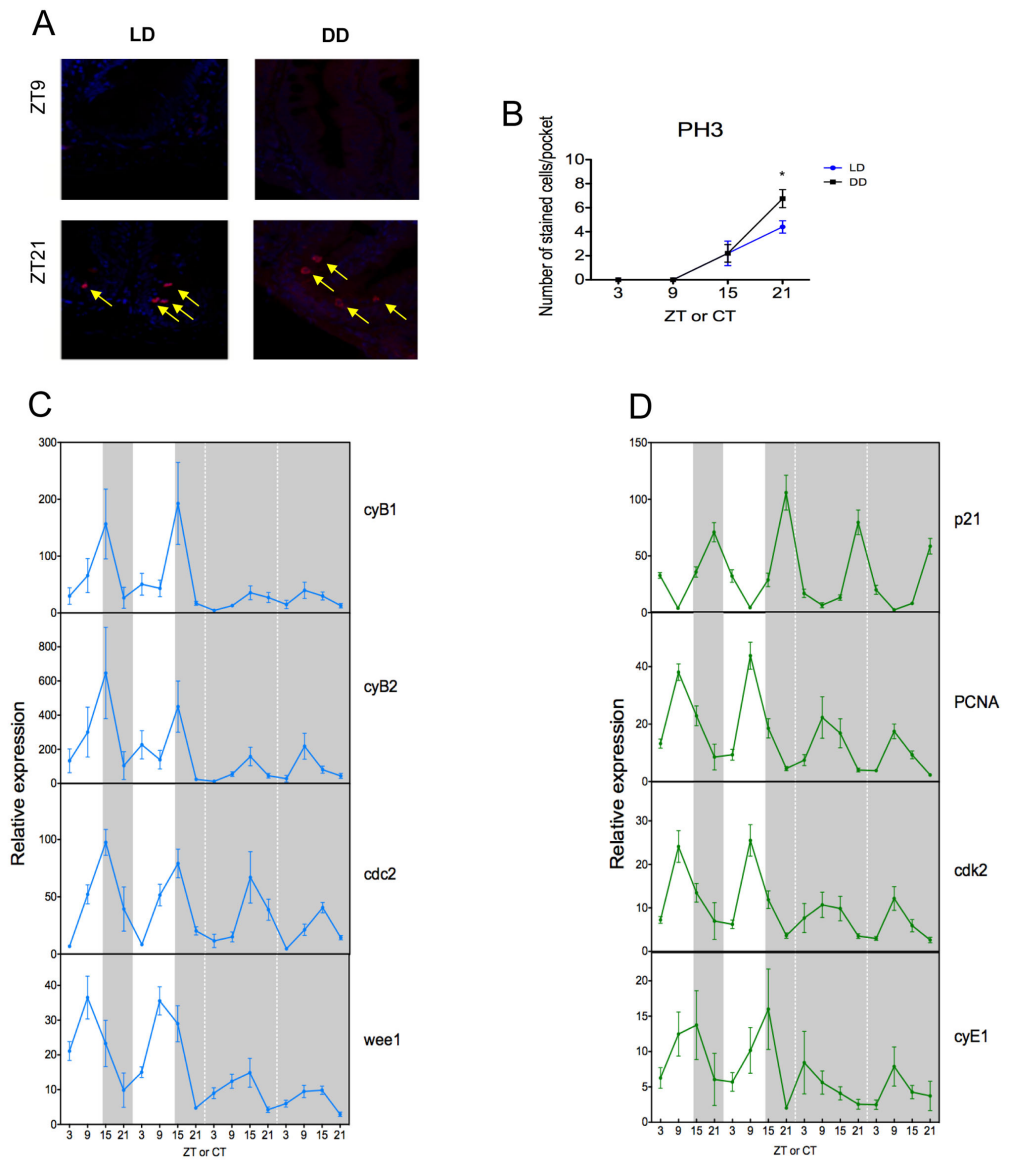
M phase is rhythmic and under circadian control. (A) In LD and DD conditions, cells stained with an anti-pH3 antibody to monitor mitosis (in red and marked by the yellow arrow) at the peak and trough time points, are localized inside the intervillus pockets of the gut. (B) Cell division is rhythmic and is exhibiting a peak of stained cells per pocket at ZT21. This rhythmicity is maintained in DD, showing that cell division is under circadian control. DAPI is used here as a nuclear counterstain. (C–D) Quantitative PCR analysis of endogenous cell cycle genes from M phase (C) and G1/S phase (D). (C) In LD, *cyB1*, *cyB2* and *cdc2* are rhythmic and peak at ZT15, whereas *wee1* peaks at ZT9. All genes continue to oscillate after entry into DD. (D) In LD, *p21*, *PCNA* and *cdk2* show a strong rhythm with peak expression at ZT9 for *PCNA* and *cdk2* and a peak at ZT21 for *p21*. In DD, all genes continue to oscillate robustly with the possible exception of *cyE1*, which shows quite variable expression even in LD. White and grey backgrounds represent light and dark phases, respectively. Cell cycle gene expression data represents the mean ± SEM from 8 fish per time point. For each time point in panel B, LD data are compared to DD using a Student’s t-test (* p<0.05). All other data are expressed as mean ± SEM.

We next examined if the intestinal pacemaker can control daily cell cycle progression, in particular, the timing of M phase or mitosis. Adult zebrafish were entrained to a LD cycle and fed twice a day, with gut samples collected and fixed at 6-hour intervals over one day. The fish were then transferred into DD, with sampling continuing for one further cycle. Sections of intestinal tissue were prepared, and cells in M phase were labeled with an antibody to phospho-Histone H3 (pH3), a well-known marker of mitosis. As can be seen in Figure 2A, cells in the intestine divide rhythmically on a LD cycle with a peak in pH3 staining (red) at ZT21 and a trough at ZT9 (full time course in Figure S1). DAPI staining (blue) labels nuclei in these sections. Dividing cells are found in the intervillus pockets, the area of the zebrafish gut known to contain the stem cell population. The mitotic rhythm observed in LD persists in DD, with a peak at CT21 (Figure 2A and 2B), demonstrating that this circadian rhythm in cell division is clock-controlled.

To further explore the mechanism by which the clock might regulate mitosis, we then measured the expression levels of key M-phase genes by qPCR. As can be seen from Figure 2C, expression of the mitotic genes *cyclin B1* (*cyB1*), *cyclin B2* (*cyB2*) and *cdc2* is rhythmic in LD, with a peak in the night at ZT15. This matches the onset of pH3 staining inside the intervillus pockets of the gut and is slightly advanced compared to the peak in pH3-positive cells, which occurs within 6 hours (Figure 2A and 2B). The *wee1* gene is also rhythmic in LD and peaks at ZT9. *Wee1* is a critical regulator of mitosis and acts by inhibiting the Cdc2-Cyclin B1 kinase (CDK1), which is required for entry into M phase. The trough of *wee1* expression occurs at ZT21 when mitosis is peaking, as one would predict for a negative regulator of mitotic entry. Rhythmic expression of these mitotic genes then continues after the animals are placed into constant darkness, but with significantly reduced amplitude, particularly for *cyB1* and *cyB2*, suggesting that these genes are only weakly clock-controlled.

Expression of genes important for the G1/S transition, including *p21*, *PCNA* and *cdk2*, are strongly rhythmic on a LD cycle (Figure 2D). The cyclin-dependent kinase inhibitor *p21* blocks entry into S phase, and its expression displays a peak at ZT21, while the peak of *PCNA* and *cdk2* expression occurs at ZT9. Following transfer into DD, *p21* gene expression remains rhythmic and peaks at CT21; *PCNA* and *cdk2* expression is also rhythmic with peak expression at CT9. The expression of *cyclin E1* (*cyE1*) appears to be highly variable, even on a LD cycle. It shows maximal expression in the early evening, which then becomes more erratic when animals are placed into constant darkness, indicating that this gene is only weakly clock-regulated, if at all. Together these results indicate that cell division and several, but not all, key regulators of M and S phases are clock-controlled in the zebrafish gut. Furthermore, there seems to be a significant 6-hour delay between the peak in mitotic gene expression and mitosis itself.

### Food deprivation has profound effects on proliferation and cell cycle gene expression in the intestine

**Figure 3:**
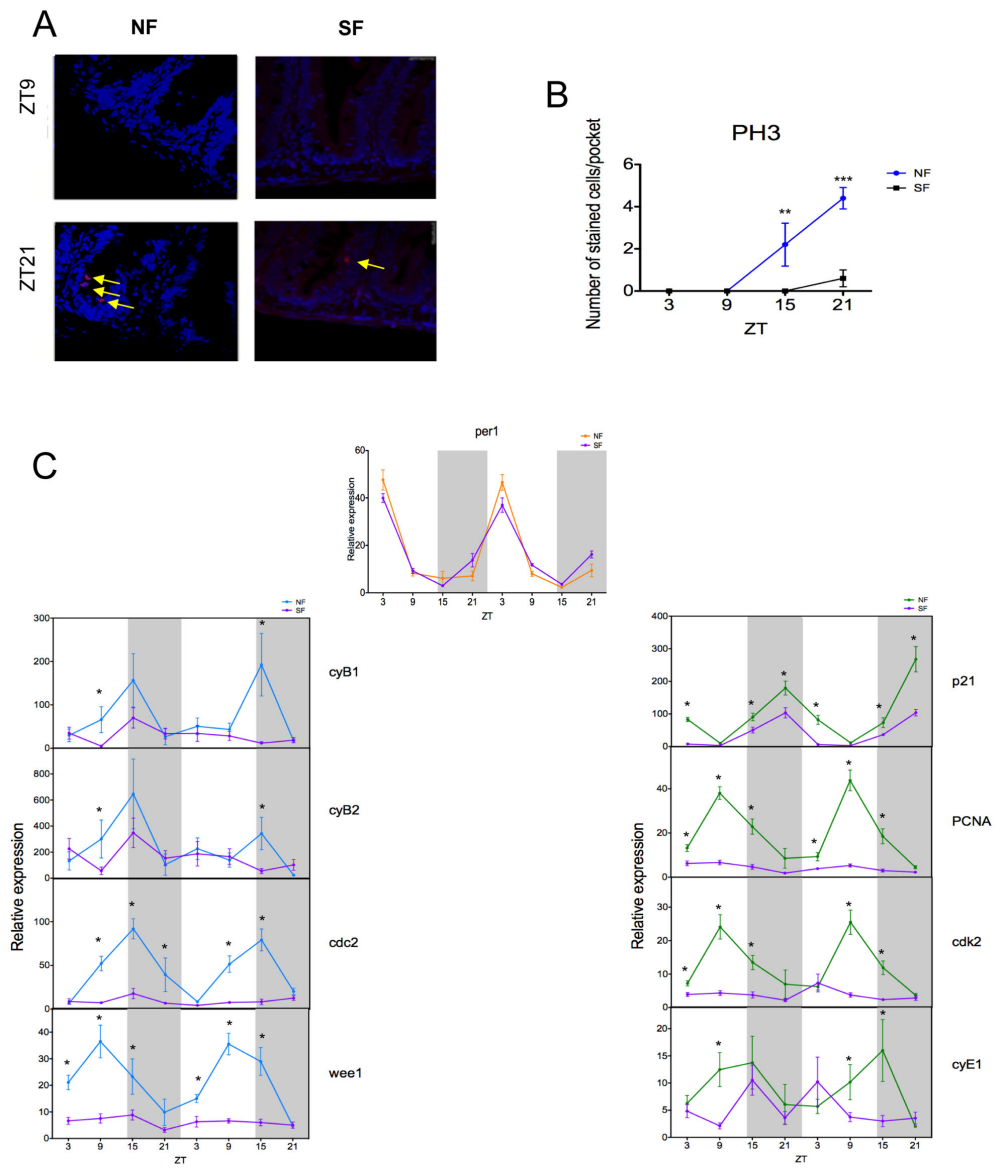
M phase is affected by starvation. (A) Cell division is rhythmic under a normal feeding schedule (NF) (peak and trough shown), but this rhythm is lost when fish are starved (SF). DAPI is used here as a nuclear counterstain. (B) Cell division is largely abolished when no food is given. (C) The *per1* rhythm is unaltered in NF and SF fish. However, all the M-phase genes studied (*cyB1*, *cyB2*, *cdc2* and *wee1*) and most G1/S-phase genes (*PCNA*, *cdk2* and *cyE1*) show reduced levels of expression, and a general loss of rhythmicity during starvation. *p21* expression is the one exception, showing a relatively small response to starvation. White and grey backgrounds represent light and dark phases, respectively. Cell cycle gene data represents the mean ± SEM from 8 to 12 fish per time point. For panels B and C, NF data are compared to SF using a Student’s t-test (* p<0.05, ** p<0.01 and *** p<0.001).

Most of the activities associated with the gut are linked to nutrition, and the digestion and absorption of food. We therefore explored the interaction between the clock, the cell cycle and food. Adult zebrafish were maintained under standard conditions on a LD cycle and fed twice a day. Food was then withheld for 2 days, and gut samples were collected every 6 hours for the following 2 days. We then compared clock and cell cycle gene expression under these starvation conditions (SF) with those obtained under normal feeding (NF) (reproduced from Figure 2). When the fish are deprived of food, there is a dramatic reduction in the number of mitotic cells at both ZT15 and ZT21, when pH3 staining is normally high (Figure 3A and 3B).

To further characterize how food deprivation affects the clock and the cell cycle, we examined the expression of the clock gene *per1*, as well as several cell cycle genes (Figure 3C). Rhythmic expression of *per1* during food deprivation is identical to that seen during normal feeding, with no significant change in phase or amplitude. This is certainly not the case for the cell cycle genes. *cdc2*, *wee1*, *PCNA* and *cdk2* show weak or no rhythmicity compared to that observed under normal feeding conditions, and, in fact, display very low levels of gene expression. *p21* continues to oscillate with similar timing to that seen under normal feeding, but with significantly reduced amplitude under starvation conditions. Thus, food deprivation does not appear to impact core clock function; it does, however, dramatically repress cell cycle gene expression and appears to uncouple the cell cycle from circadian clock control.

### Timed feeding drives circadian rhythms in clock and cell cycle gene expression

**Figure 4:**
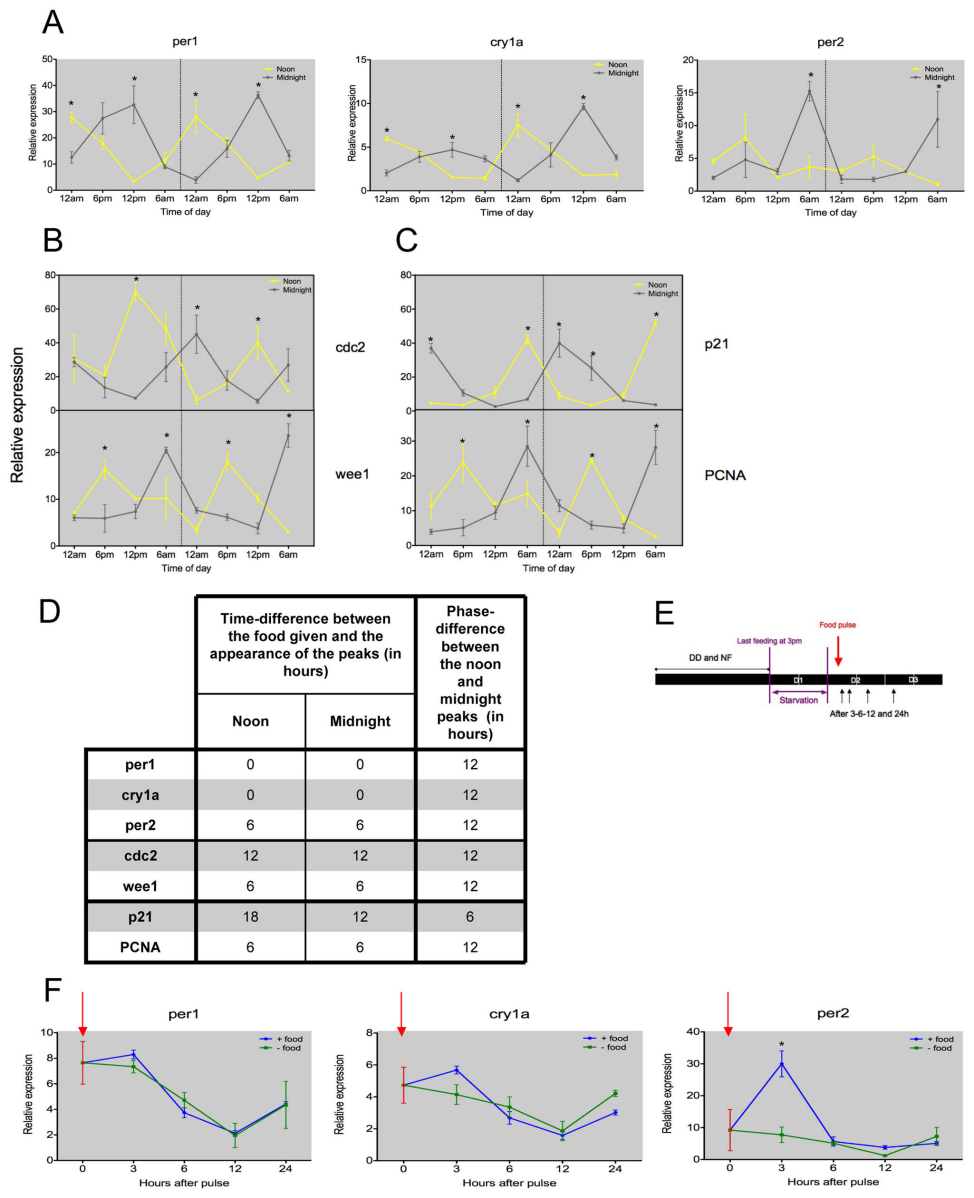
Intestinal circadian clock and cell cycle genes are food-entrainable in zebrafish. (A) In DD, clock genes *per1*, *cry1a* and *per2* are rhythmically expressed during restricted feeding, when food is provided at noon or midnight. The rhythms in clock gene expression retain a stable phase relationship between the two opposite feeding schedules. (B–C) Key cell cycle regulators show corresponding entrainment to the two opposite feeding regimes. *cdc2* peaks are observed at midnight for noon fed animals and at midday for the midnight fed animals. *wee1* expression shows peak values 6 hours after the feeding time. (D) The table illustrates the time difference between the feeding regime, noon or midnight, and peak expression for all the genes studied, as well as the phase difference between the two experiments. (E) A schematic of the food pulse experimental design. (F) There is no acute effect of feeding for *per1* and *cry1a* expression. In contrast, *per2* expression after 3h is increased compared to the unfed control. Red arrow represents timing of the food pulse. Grey backgrounds represent constant dark conditions. Data represents the mean ± SEM from 3 or 4 fish per time point. For panels A, B and C, samples collected at noon are compared to those collected at midnight using a Student’s t-test. For panel D, at each time point after the food pulse, a Student’s t-test is employed to compare the two conditions (* represents a significant statistical difference of p<0.05).

We next asked whether different feeding schedules would affect the interaction between the clock and the cell cycle. To address this question, two groups of fish were maintained in DD for one week. During this period, one group of fish was fed at noon and the other group was fed 12 hours later at midnight. Intestinal tissue was then dissected every 6 hours on days 7 and 8 of this timed feeding regime. The expression levels of *per1* were assessed by qPCR. *per1* showed strong rhythmic expression in both groups of fish and peaked at ZT0, defined here as the time of feeding (Figure 4A). The intestinal clock was therefore perfectly out of phase between the two groups on opposite feeding schedules (Figure 4D). Interestingly, when we examined the light-inducible genes, *cry1a* and *per2*, they were both rhythmically expressed, but peaked at different times. The peak in *cry1a* occurred at ZT0, similar to *per1*, whereas the peak in *per2* was at ZT6. This peak in *per2* expression lags the feeding time by approximately 6 hours, which is a rather unexpected phase relationship to the feeding schedule and could reflect the fact that this gene is, in fact, acutely induced by food (see below). Regardless, it is clear that timed feeding can entrain the zebrafish intestinal circadian clock.

We then compared cell cycle gene expression under the two feeding regimes (Figure 4B and 4C). *cdc2*, *wee1*, *p21* and *PCNA* show clear entrainment to timed feeding. *cdc2* expression peaks 12 hours after feeding, whereas *wee1* peaks within 6 hours after feeding. Interestingly, these mitotic genes maintain the same phase relationship as on a LD cycle. Of the genes involved in S-phase regulation, *PCNA* expression is clearly entrained, with peak expression occurring 6 hours after feeding. *p21*, however, shows a much more complex entrainment response, with peak expression occurring some 12 hours post feeding, when feeding occurs at midnight, but then 18 hours later when feeding occurs at noon. In other words, the opposite food schedules do not entrain expression of this particular gene in an equal and opposite manner. The relative phasing of these genes to the feeding regime and to each other is summarized in Figure 4D.

Entrainment to a feeding schedule clearly occurs in terms of the intestinal circadian clock, as well as some cell cycle components. The mechanism by which light is thought to phase shift the zebrafish circadian pacemaker is somewhat better understood. The acute light induction of *per2* and *cry1a*, amongst other factors, are key to this event [20–22]. We therefore looked at the acute response of these two genes, and *per1*, following a pulse of food. Fish entrained on a 14:10 light-dark cycle and fed twice a day were transferred into DD with the same feeding schedule. 24h before sampling, the fish were starved, at which point one group of individuals were given a single pulse of food at noon on the subsequent day. Control animals were not given this food pulse, and were maintained in DD. Intestinal tissue was sampled at 0, 3, 6, 12 and 24 hours after the food pulse (Figure 4E). There was no acute effect of the food pulse for *per1* and *cry1a* expression (Figure 4F). In contrast, *per2* exhibited a high level of expression 3 hours after feeding, compared to the control group without food. Following this peak in expression, *per2* levels returned to the same, basal level seen in unfed fish. Though this is far from proof of a role for *per2* in food entrainment, it is interesting that at least one common element is induced in both food and light entrainment pathways.

### Random feeding in LD and DD alters cell cycle gene oscillations

It is clear that either a LD cycle or a rhythmic feeding regime can entrain both the intestinal clock and cell cycle gene expression rhythms. In a “real world” situation, obviously, both light and feeding signals must interact to produce stable oscillator phase and regulate timing of downstream processes. Feeding cues are far less predictable than the environmental LD cycle, but does this, in fact, influence or disrupt normal clock entrainment, especially in a tissue like the intestine? To explore this issue, we examined the impact of random feeding on clock and cell cycle gene expression rhythms under light entrained and free-running conditions. For this purpose, adult zebrafish were entrained on a LD cycle and then fed at random times for one week in either LD or DD conditions prior to sampling. Expression levels for clock and cell cycle genes were then compared between randomly fed fish (RF) and those fed twice a day (NF) in LD or once a day in DD (reproduced from Figures 2 and 4, respectively).

**Figure 5:**
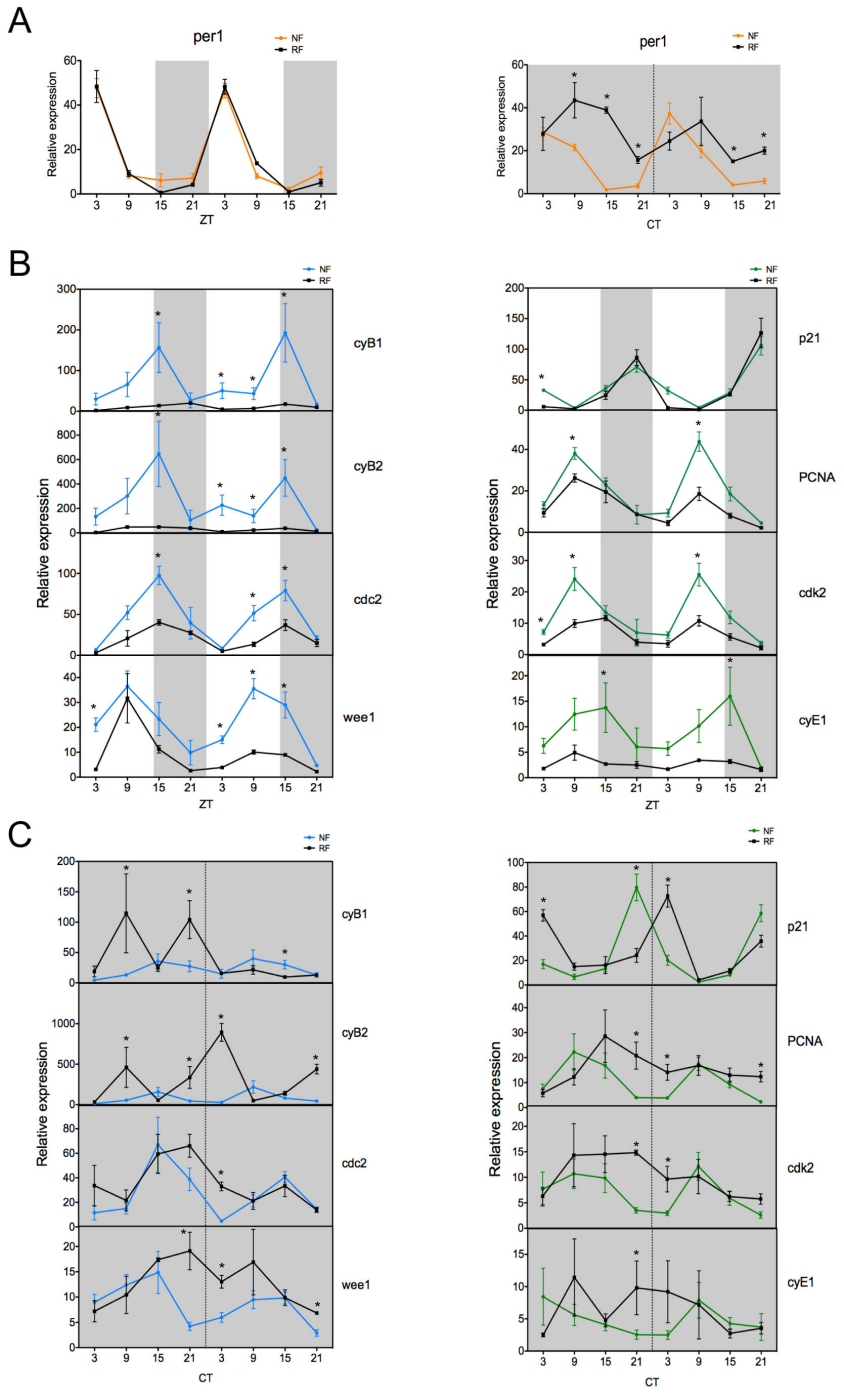
Random feeding in LD and DD alters cell cycle gene oscillations. (A) Entrained to a LD cycle, the rhythm and amplitude of *per1* expression is not altered by the feeding regime, either normal (NF) or random (RF). In DD and NF, *per1* is rhythmic, but under RF and DD conditions, *per1* ceases to show a robust, precise daily rhythm. (B) *Cyclin* gene expression (*cyB1*, *cyB2* and *cyE1*) shows a large reduction in the level of expression and very shallow oscillations in RF compared to NF. Under RF, expression of *cdc2*, *wee1*, *PCNA* and *cdk2* displays a similar rhythmicity to NF, but with significantly reduced amplitude. *p21* expression is not altered by the feeding regime. (C) When fish free-run in DD and are fed at random times, all the genes studied (*cyB1*, *cyB2*, *cdc2*, *wee1*, *PCNA*, *cdk2* and *cyE1*), except *p21*, display a disrupted profile compared to NF. No clear rhythmicity is observed. White and grey backgrounds represent light and dark phases, respectively. Uninterrupted grey backgrounds represent constant dark condition. Data represents the mean ± SEM from 3 or 4 fish per time point. For panels A, B and C, NF data are compared to RF at each time point using a Student’s t-test (* represents a significant statistical difference of p<0.05).

An examination of *per1* expression on a LD cycle under normal (NF) and random (RF) feeding regimes shows how light dominates the entrainment of the intestinal circadian pacemaker (Figure 5A). Random feeding does not impact clock entrainment in terms of either circadian phase, or the amplitude of the rhythm. Under DD conditions, timed feeding entrains the circadian clock, as shown in Figure 5A (right panel, orange line). The removal of both entraining cues (random feeding in DD), not surprisingly, leads to a much more temporally chaotic situation (Figure 5A, right panel, black line). Any residual rhythm is greatly damped, with a lack of precise daily timing.

What are the consequences of the above experimental regimes on cell cycle gene oscillations? Again, not surprisingly, under normal LD entrained conditions and rhythmic feeding, there are clear circadian rhythms for all cell cycle genes examined (Figure 5B). However, the results under LD with random feeding were more unpredicted. The rhythmic expression of the *cyclin* genes (*B1*, *B2* and *E1*) normally seen under LD conditions is effectively abolished, with general expression falling to very low levels (Figure 5B). In this way, random feeding resembles a starvation response and clearly uncouples a fully entrained molecular clock from driving downstream rhythmicity. With the exception of *p21* gene expression, which is not significantly altered by random feeding under LD entrainment, all of the remaining clock-regulated cell cycle genes (*cdc2*, *wee1*, *PCNA*, and *cdk2*) show a dramatic reduction in the amplitude of rhythmic expression, demonstrating that the rhythmic feeding signal strongly consolidates the link between core clock function and the regulation of rhythmic downstream gene expression in the intestine. In the absence of light, but in the presence of normal scheduled feeding, the cell cycle genes previously described in Figure 4 as food entrainable, still show timed gene expression profiles (Figure 5C). In general, however, this is not true for expression of the *cyclin* genes, which does not show robust, entrained rhythms to feeding schedules. When both entraining cues are absent (light and timed feeding), then cell cycle gene expression tends to follow the disrupted clock profile and does not show any clear rhythmicity, with the interesting exception of the *p21* gene (Figure 5C).

## Discussion

The circadian system in zebrafish has long been recognized as being highly decentralized, with circadian pacemakers in numerous tissues, which are themselves directly light responsive [14]. In this study, we have extended this observation to the zebrafish intestine, showing that the gut generates robust oscillations in *per1* expression *in vivo*, which free-run under constant dark conditions (Figure 1A). This tissue also responds to acute light pulses, with the induction of *per2* and *cry1a* gene expression that has been previously reported for zebrafish cell lines and embryos (Figure 1B). The use of the *per3-luciferase* transgenic fish allowed us to confirm that this circadian pacemaker is endogenous to this tissue and continues to function over at least 11 days in culture (Figure 1C). The intestine is directly light responsive *in vitro* as well, as this rhythm in *per3* can be re-entrained to an altered, inverted light-dark cycle applied directly to the cultured tissue (Figure 1D).

One major feature of intestinal tissue is the requirement for regular and controlled cell renewal. The very nature of its role in the digestion and absorption of nutrients places considerable “pressure” on the cells that line the epithelium of the gut. Consequently, sustained cell proliferation is essential for this tissue to perform this digestive function. Stem cell populations at the base of the intestine between villi (crypts in mammals, intervillus pockets in zebrafish) lead to the production of new cells, which then differentiate as they move up along the length of the villi. Old cells are then shed at the end of these villi through the process of apoptosis[15]. . Cell division in the mammalian gut has long been known to be under the control of the circadian clock [24]. In this study, we have shown that this is also true for the zebrafish intestine, with the timing of mitosis being under clock control. These results are similar to those that we have recently shown both in developing zebrafish larvae and zebrafish embryonic cell lines [25,26]. Fundamentally, circadian clock control of the cell cycle occurs at the cellular level, although tissue level and systemic cues very likely play a role in the control of this process. An example of the additional level of complexity of this regulation can be seen from our food deprivation experiments. If zebrafish are not fed for two days prior to sampling, there is a significant reduction in the number of dividing cells, and the cell cycle is effectively inactivated. Interestingly, there are no measurable consequences on circadian clock gene expression, and the clock appears to function normally. Following this level of starvation, there is a dramatic impact, however, on cell cycle gene expression. All of the cell cycle genes examined, with the exception of *p21*, show a loss of clock control. This of course corresponds to the general large-scale reduction in cell division seen with starvation. The circadian clock and the regulation of cell cycle gene expression are effectively uncoupled in the absence of adequate feeding. It will be very interesting to see in future studies if starvation has the same impact on the level of cell division within intestinal tumors. If so, the careful control of nutrition and timing of meals could represent a useful tool in maximizing the efficacy of chemotherapeutic treatments. The ability to “shut down” cell cycle progression in healthy cells by controlling diet could prove to be a useful method to reduce the levels of undesirable cell death during a program of chemotherapy, focusing the impact of these drugs mainly on the malignant cell populations.

When timed feeding is the only entraining cue (in constant darkness), this feeding regime appears to entrain the circadian pacemaker within the gut, although to confirm this, persistence of *per1* rhythms must be measured after entrainment during the first day of food deprivation. The phase relationship between clock and feeding time is such that *per1* expression peaks precisely at the time of feeding. We do not believe that this represents a food-driven response or masking, as expression of *per1* begins to increase prior to, or in anticipation of, the feeding time. This is clearly not the case for *per2*, which following feeding shows a 6-hour delay in peak expression and a rhythm that is somewhat less robust than for *per1*. We believe that *per2* responds directly to the feeding event, as its expression is transiently increased following a pulse of food (Figure 4F), unlike that for *per1* and *cry1a*. *per2* has been strongly implicated in the input pathway for light entrainment in zebrafish. Though these results do not prove a similar role for *per2* in food entrainment, they certainly support this hypothesis. Key components of the cell cycle also show clear entrainment to timed feeding, with *p21*, *PCNA*, *cdc2* and *wee1* all showing strong rhythms that, with the exception of *p21*, maintain a stable phase relationship to the feeding schedule. Entrainment of the circadian clock by rhythmic feeding can, for the most part, regulate the timing of cell cycle gene expression in the intestine. However, we have not yet eliminated the possibility that the rhythmic feeding regime itself could be directly driving the daily oscillations in the cell cycle, without the need of a circadian pacemaker. Our experiments would ideally be repeated in clock mutant/arrhythmic animals, lacking a functional circadian pacemaker, and the result of rhythmic feeding on the cell cycle could then be determined. As reduced feeding dramatically reduces the amount of cell division, and cell cycle gene oscillations, it is not possible to enter free-running conditions following a timed feeding regime.

Circadian food entrainment in mammals is quite complex, and food-anticipatory activity (FAA) has been shown to be under circadian clock control, but does not require a functional circadian pacemaker within the SCN [27]. The search for a localized food entrainable oscillator (FEO) in the mammalian brain has so far been inconclusive, and this process could be the product of a more diffuse neural network, rather than a discreet nucleus. It is also clear, however, that food can directly entrain the clocks within peripheral tissues, such as the liver and stomach. This has been best demonstrated in rodents when restricted feeding has been provided to animals in their inactive phase, out of synchrony with their normal activity rhythms. Under such circumstances, the phase of the clock in the liver will re-entrain to the feeding cue, while the “master” clock in the SCN will remain entrained to the LD cycle. In the case of zebrafish, the circadian system is highly decentralized, with each tissue being directly light responsive. In the case of food entrainment of the gut, therefore, our working hypothesis is that this tissue clock will itself be directly entrained to the feeding cue, without the need for central pacemakers. Evidence for this and the mechanism by which this occurs is not clear at this time, and further studies will be required to exclude the presence of a possible central FEO.

The entrainment of clocks and their rhythmic outputs is much more complex in a “real world” setting, as animals are exposed to a number of environmental cues that are able to influence the circadian system. How do light and food then interact to regulate circadian phase and the timing of cell cycle events, particularly in a tissue such as the intestine? In zebrafish, the answer is clear, with light acting as the dominant entrainment cue. Fish on a light-dark cycle maintain precise and identical timing of molecular clock oscillations whether they are exposed to normal, diurnal feeding or randomized feeding schedules, since random feeding schedules do not impact on the light entrained circadian pacemaker in the intestine. Normal, noontime feeding in constant darkness can also entrain the circadian clock, with peak *per1* expression occurring at the time of feeding or ZTO. Fish are mostly active during the day, and not surprisingly, tend to eat at this time. Normal feeding regimes and light both act to set the intestinal pacemaker, “working together” to consolidate optimal clock entrainment.

The strong clock entrainment seen under light-dark conditions and rhythmic feeding, not surprisingly, leads to robust oscillations in cell cycle gene expression in the intestine. Rather more surprisingly, random feeding has an impact on the cell cycle quite similar to that of starvation. The levels of *cyclin* gene expression are dramatically reduced and the amplitude of rhythmic gene expression is much lower, as can be seen for *cdc2* and *wee1* oscillations. Curiously, *p21* expression is not influenced by this random feeding regime, and always retains robust oscillations linked to the light-dark cycle. It is clear from these results that the rhythmic presence of food is essential for the clock to be effectively coupled to the control of cell cycle timing in the intestine, and for the adequate expression of key cell cycle genes, especially *cyclin B1*, *B2* and *E1*. The light-dark cycle is sufficient for the normal entrainment of the intestinal circadian clock, but without rhythmic feeding, cell cycle gene expression is far from optimally entrained, and intestinal physiology may be compromised.

## Materials and Methods

### Fish care

Adult wild type (AB-TL) zebrafish (*Danio rerio*) were raised at 28°C in the fish facility of University College London according to standard procedures [28]. All animals were held in a Home Office approved animal facility and in accordance with Home Office regulations regarding animal maintenance and care. Animal handling has been approved by a UCL ethics committee and meets all of the requirements of the Animal Welfare Act of 2006. Individual experiments were performed under animal license number PIL 70/23714. Animals were sacrificed in accordance with Schedule 1 of the Animal Welfare Act of 2006, to ensure that minimal suffering or discomfort was inflicted on all animals in this study. Fish tanks were kept in light tight cabinets, where the light cycle was 14 hours light, 10 hours dark (14L:10D), unless indicated otherwise, with an average intensity of 800 µW/cm^2^. The fish were fed twice a day, unless stated otherwise (see below).

### Experimental design

In all experiments, fish were fed a standard commercial diet (Hikari tropical and SAFE Caviar 500-800 micro pellets) added to fresh brine shrimp. Whole intestine was dissected from adult fish and harvested according to the specific experimental design (see below).

#### Experiment 1: Normal feeding

Adult zebrafish were maintained in a light cabinet in separate tanks, each tank corresponding to one time point, and fed twice daily. For the first 2 days, fish were exposed to a LD cycle (14L:10D) and then transferred into DD for an additional 2 days. The fish were sacrificed every 6h over 4 days.

#### Experiment 2: Starvation

Adult zebrafish were maintained in 8 separate tanks, each tank corresponding to one time point, in a light cabinet on a normal light-dark cycle (14L:10D) and fed twice a day. Two days before the sampling, the fish were starved and then sacrificed every 6h over the subsequent 2 days.

#### Experiment 3: Food entrainment

Adult zebrafish were maintained for one week in DD in two separate cabinets, 8 tanks each. In the first cabinet, fish were fed once a day at noon and in the second cabinet, once a day at midnight for one week. Subsequently, whole intestine was harvested every 6h for 2 days.

For the food pulse experiment, fish fed twice a day were entrained on a LD cycle (14L:10D) and then transferred to DD for one week. Fish were then starved for one day, and a food pulse was given at noon of the following day. Gut samples were collected at 3, 6, 12 and 24h after the food pulse.

#### Experiment 4: Random feeding

Adult zebrafish were maintained for one week in separate tanks, each tank corresponding to one time point. These tanks were housed in 2 separate cabinets, one in LD (14L:10D) and one in DD. Fish were fed on a randomized feeding schedule both day and night. Whole intestine was collected every 6h for 2 days.

### Statistical analysis

All data presented in this manuscript are plotted as mean ± SEM and analysed by a two-tailed Student’s t-test. A p-value of less than 0.05 was considered significant (* p<0.05, ** p<0.01 and *** p<0.001).

### Quantitative PCR

Whole zebrafish intestine was collected at the indicated time points in Trizol® (Invitrogen), homogenized and then stored at -20°C. RNA extraction was performed according to manufacturer’s instructions (Invitrogen). cDNA was synthesized from 1 µg of total RNA using SuperScript II Reverse Transcriptase (Invitrogen). Quantitative PCR was performed using SYBR Green Jumpstart Taq Ready Mix (Sigma-Aldrich) and gene-specific primers on a Mastercycler Ep Realplex^2^ (Eppendorf). The relative levels for each RNA were calculated by the 2^-∆∆Ct^ method. Expression levels were normalized to a reference gene (ribosomal protein L13α) and then to a single sample with the highest ∆Ct value. Each sample was analyzed in triplicate. See Table S1 for a list of qPCR primers used in this study.

### Bioluminescence assays

Bioluminescent signals from *per3-luciferase* transgenic zebrafish [23] were monitored on a Packard TopCount NXT scintillation counter. Sections of transgenic gut were placed into a 96-well plate containing L15 media with 15% Fetal Calf Serum (FCS), penicillin/streptomycin (100 U/ml), gentamycin (50 µg/ml) and 0.5 mM luciferin (Promega). The plates for Figure 1C were maintained at 28^°^C on a LD cycle (14L:10D) for 6 days, then transferred into DD for 4 days and back into LD for an additional 4 days. The plates for Figure 1D were maintained at 28^°^C on a LD cycle (14L:10D) for 7 days, then transferred into DL (10D:14L) for 6 days.

### Tissue fixation and cryosectioning

Gut samples were fixed overnight in a 4% paraformaldehyde (PFA) solution in 0.1 M Phosphate Buffer (PB) at 4°C. After phosphate-buffered saline (PBS) washes, fixed tissues were incubated overnight in 30% sucrose in 0.1 M PB at 4°C, then embedded in OCT (Sakura) and frozen at -80°C. Tissue was cryosectioned at a thickness of 12 µm using a Leica cryostat (CM1900UV). Tissues in OCT moulds and sections were stored at -80°C.

### Phospho-Histone H3 immunohistochemistry

Cryosections were fixed for 10 min at room temperature in a 4% PFA solution in 0.1 M PB, washed with PBS-0.1 % Tween (PBT), and then blocked at RT with 1% BSA, 1% Triton X-100 in PBT. An anti-phospho-histone H3 antibody (1:500, Millipore) was applied overnight in the blocking solution at 4°C. After PBT washes, an anti-rabbit Alexa Fluor 568 secondary antibody (1:1000, Invitrogen) was applied for 30 min and rinsed with PBS before counterstaining for 5 min with DAPI and mounting in Vectashield (Vector Laboratories).

## Supporting Information

Figure S1 **Mitosis in the zebrafish intestine is rhythmic and under circadian control, but is abolished during starvation.** Extended time course of cell division, using a pH3 antibody as a mitotic marker, for fish fed twice a day and entrained to a LD cycle then transferred into DD, as well as for starved fish (SF) entrained to a LD cycle. Gut samples were collected every 6h for the three conditions. In LD, cells divide rhythmically with a peak at ZT21; when the fish are free-running in DD, this rhythmicity is maintained. When the fish are starved, no cell division is observed.
(TIF)

Table S1 **List of primers used in the qPCR analysis.**
(TIF)
