# Supplementary figures and images for: Circadian Clock Regulation of the Cell Cycle in the Zebrafish Intestine
Source: PLoS One. 2013 Aug 27;8(8):e73209. doi: 10.1371/journal.pone.0073209 (PMC3754960; doi:10.1371/journal.pone.0073209)

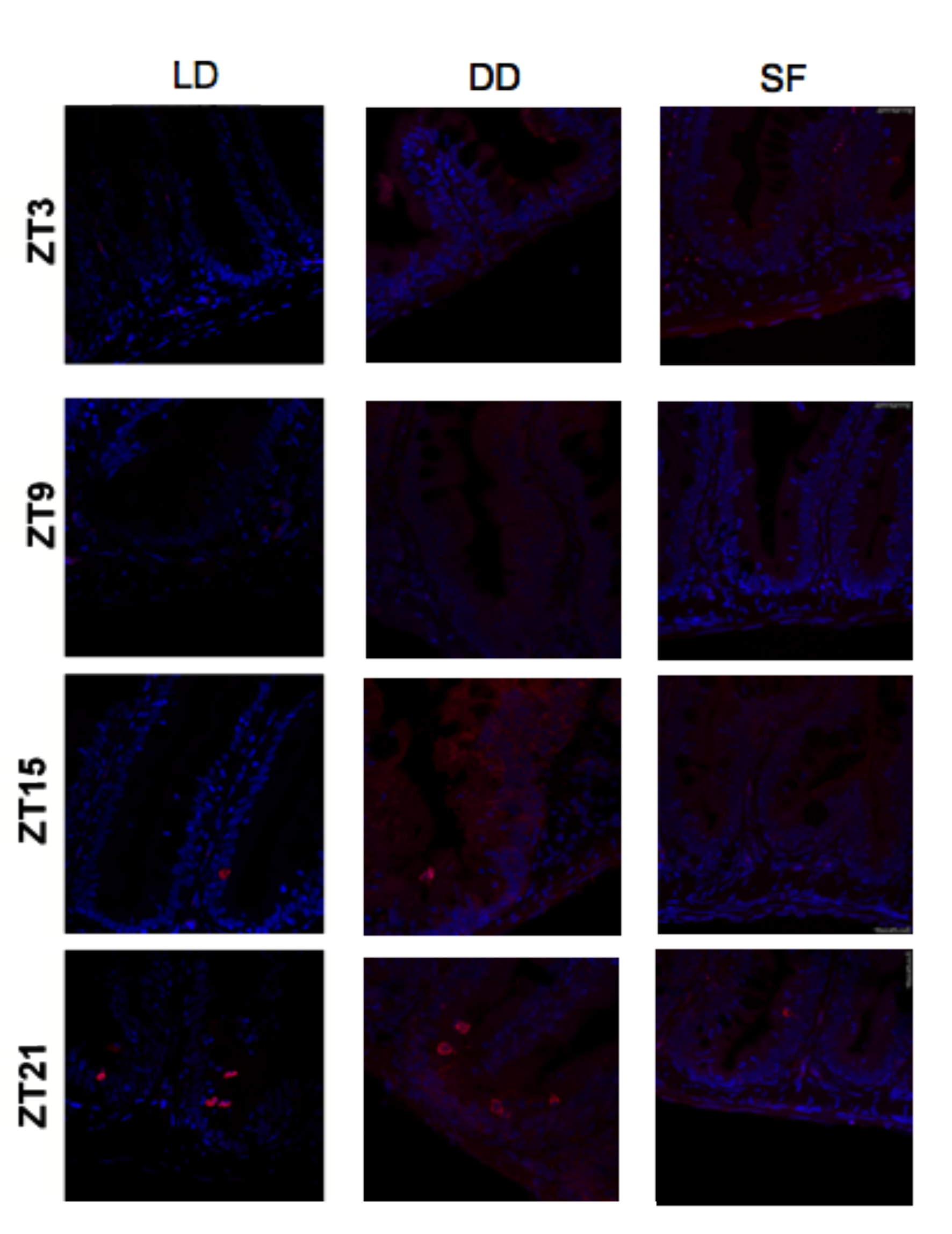

Supplement: Figure S1 — Mitosis in the zebrafish intestine is rhythmic and under circadian control, but is abolished during starvation. Extended time course of cell division, using a pH3 antibody as a mitotic marker, for fish fed twice a day and entrained to a LD cycle then transferred into DD, as well as for starved fish (SF) entrained to a LD cycle. Gut samples were collected every 6h for the three conditions. In LD, cells divide rhythmically with a peak at ZT21; when the fish are free-running in DD, this rhythmicity is maintained. When the fish are starved, no cell division is observed. (TIF) [file pone.0073209.s001.tif]

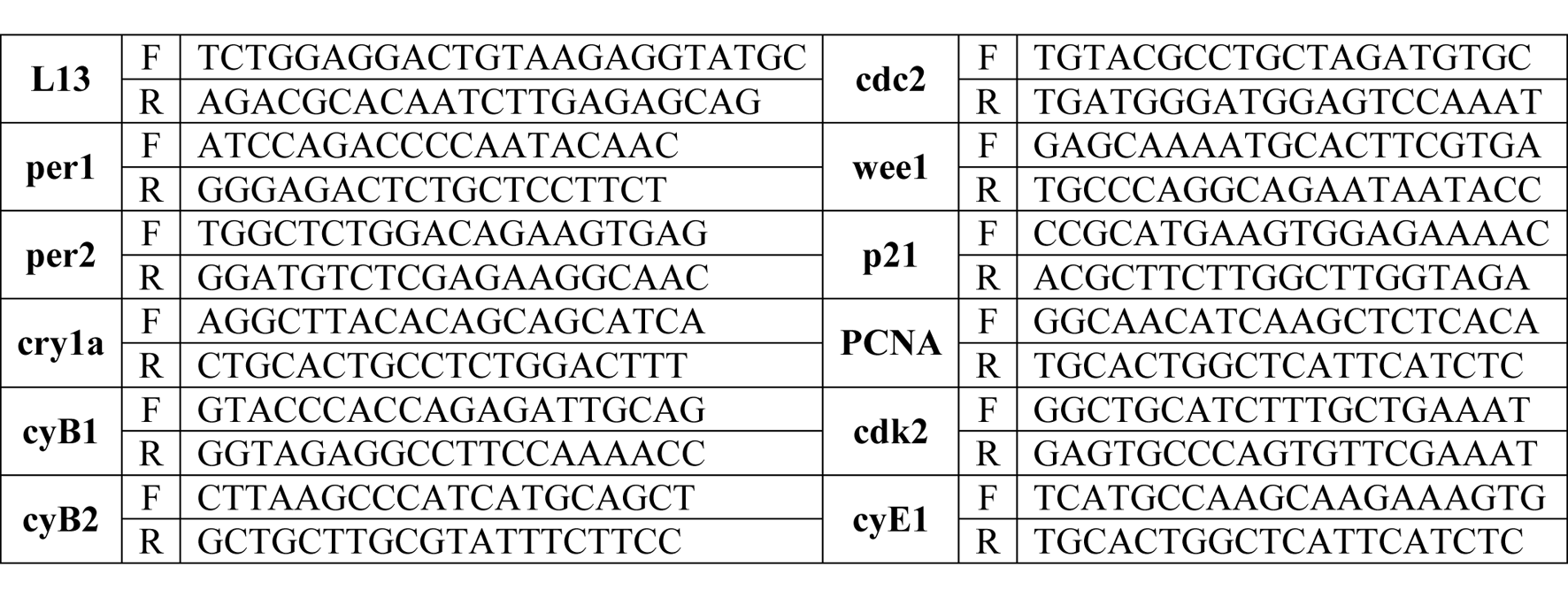

Supplement: Table S1 — List of primers used in the qPCR analysis. (TIF) [file pone.0073209.s002.tif]
